# Feasibility and preliminary outcomes of a peer-led diabetes self-management support program for black men: the michigan men’s diabetes project (MenD II)

**DOI:** 10.3389/fcdhc.2026.1755183

**Published:** 2026-05-26

**Authors:** Jaclynn M. Hawkins, Alana M. Ewen, Maricruz Moya, Katherine A. Kloss, Hannah Burgess, Srijani Sengupta, Martha Funnell, Robin Nwankwo, Gretchen Piatt

**Affiliations:** 1 School of Social Work, University of Michigan, Ann Arbor, MI, United States; 2 Department of Learning Health Sciences, School of Medicine, University of Michigan, Ann Arbor, MI, United States; 3 Department of Behavioral and Community Health, School of Public Health, University of Maryland, College Park, MD, United States; 4 Department of Health Behavior & Health Equity, School of Public Health, University of Michigan, Ann Arbor, MI, United States

**Keywords:** black men, diabetes self-management, health disparities, peer support, pilot study, type 2 diabetes

## Abstract

**Background:**

Black men face disproportionately high rates of type 2 diabetes (T2D), yet are underrepresented in diabetes self-management support (DSMS) interventions. Peer-led DSMS (PLDSMS) shows promise for minoritized populations, but few programs target Black men specifically.

**Objective:**

This pilot study examined feasibility and preliminary effectiveness of a culturally adapted, PLDSMS intervention for Black men with T2D in Metro Detroit.

**Methods:**

We conducted a 15-month randomized pilot trial (N = 48) comparing PLDSMS plus DSMES to DSMES alone (enhanced usual care). PLDSMS included monthly peer-led virtual sessions for six months post-DSME, plus six months of optional support. Primary outcomes included HbA1c and perceived diabetes self-management (PDSMS). Secondary outcomes included diabetes distress and social support at baseline, 3, 9, and 15 months.

**Results:**

Participants (mean age=63 years) demonstrated successful recruitment (N = 60) and high retention (>80% at 15 months). No between-group differences in HbA1c emerged (p=0.32). Perceived self-management showed significant group-by-time interaction (p=0.03); the control group scored 5.36 points higher at 3 months (p=0.02), but differences were not sustained at 9 months (p=0.67). Diabetes distress and social support did not differ between groups, though provider-related distress declined significantly across both arms (B=-0.54, p=0.001).

**Conclusion:**

This pilot demonstrates feasibility of a PLDSMS program for Black men. Glycemic control remained stable, and although between-group differences in perceived self-management were not sustained over time, findings are consistent with the hypothesis that peer-led support may help sustain self-management gains as the initial benefits of DSME alone begin to wane. Future fully powered trials are needed to test this hypothesis.

## 1 Introduction

Type 2 diabetes (T2D) is a prevalent chronic disease in the United States, affecting more than 38 million people, roughly 1 in 10 Americans, with 90–95% of cases being type 2 (1). This burden is not evenly distributed: Black men are nearly twice as likely to develop T2D as non-Hispanic White men and are about twice as likely to die from T2D-related complications (2). In Michigan, the impact of diabetes mirrors these national trends. Approximately 1.16 million Michigan residents (11.6% of the population) have diabetes, making it the state’s 8th leading cause of death (3). Black men in Michigan bear a disproportionate share of this burden, they are more frequently diagnosed with T2D and have significantly higher mortality rates. For instance, the age-adjusted T2D death rate among Black males in Michigan is about 50.0 per 100,000, far above the rate of 32.5 per 100,000 observed in White males (3). This epidemiologic context underscores the importance of improving T2D outcomes among Black men at both national and state levels.

Despite the high prevalence of T2D, Black men often experience worse diabetes-related health outcomes and face unique barriers to care. Once diagnosed, Black men tend to have suboptimal glycemic management compared to non-Hispanic White men, which elevates their risk of developing serious diabetes-related complications (4). A growing body of research attributes some of these disparities to sociocultural factors, particularly gender norms and expectations (5–7). Many Black men grow up with ideals of toughness, emotional restraint, and self-reliance that can conflict with engaging in health care and self-management behaviors (5, 6). In practice, these norms can discourage Black men from seeking medical help or following professional health advice, a phenomenon sometimes referred to as the “superman syndrome,” wherein men avoid appearing vulnerable by forgoing care (5, 6, 8, 9). Such gendered expectations create barriers to asking for support with lifestyle changes and may foster mistrust with the healthcare system or limited communication with providers (5, 6). These challenges contribute to Black men’s lower engagement in recommended diabetes self-management programs.

Evidence suggests that Black men are less likely to consistently perform T2D self-care behaviors (e.g., healthy eating, regular blood glucose monitoring) compared to non-Hispanic White men and Black women (5, 6). They are also more prone to certain risk behaviors, such as smoking and excessive alcohol use due to life stressors rooted in systemic inequities, which further complicate diabetes management (10–12). Collectively, these factors help explain why Black men with T2D suffer higher rates of diabetes-related complications, distress and mortality. Addressing T2D in this population requires not only managing the clinical aspects of the disease but also overcoming the social and behavioral barriers rooted in masculine role norms and past experiences with the health system.

One promising strategy to improve outcomes in this group is the use of peer-led diabetes self-management education and support. Diabetes Self-Management Education (DSME) provides patients with the knowledge and skills for day-to-day T2D care, and ongoing Diabetes Self-Management Support (DSMS) offers continued help with applying those skills and coping with challenges (13, 14). National standards affirm that DSME combined with ongoing DSMS leads to better glycemic control and self-care outcomes than education alone (14). However, traditional DSMES programs are often clinic-based and led by health professionals, and Black men have historically been less likely to enroll in or benefit from these standard programs, evidenced by lower rates of engagement, recruitment, and retention (7, 28). Peer support models, where trained community members or fellow patients deliver DSMES, have shown success in improving T2D management in racially marginalized communities through culturally relevant education, goal-setting, problem-solving, and social support (16).

Black men appear to respond especially well to interventions delivered by peers who share their lived experience. Emerging research indicates that gender-concordant, male-to-male programs can reduce the stigma men often feel in seeking help and foster more open communication about health (17–19). Using male coaches or facilitators has been effective in engaging men in other health contexts, such as hypertension control and cancer screening in Black barbershops (20–24). Despite these preferences, most diabetes peer-support interventions to date have been led by women and have predominantly female participants (25–27); Black men are less likely to participate in T2D-related intervention studies compared to non-Hispanic White men (28). This highlights a critical gap in T2D care outreach. Tailoring peer-led diabetes self-management support (PLDSMS) specifically for Black men, by recruiting male peer leaders and addressing topics through a masculine cultural lens, is posited to improve both engagement and outcomes (29).

The MenD intervention was deliberately grounded in theoretical frameworks that emphasize patient autonomy and empowerment. Self-Determination Theory (SDT) and the Patient Empowerment model of diabetes care informed the design of the peer support program (30–34). SDT posits that individuals are more motivated and successful in behavior change when their basic psychological needs, autonomy, competence, and relatedness, are supported (32, 34). Prior studies have shown that diabetes education incorporating autonomy support and empowerment principles leads to improved self-care behaviors and clinical outcomes (35–37). By leveraging SDT and the Patient Empowerment model, the MenD intervention aimed to engage Black men in a manner that resonates with their values and ultimately fosters sustained, intrinsically motivated self-management.

Considering the above context, our team developed the Michigan Men’s Diabetes (MenD) Project as a series of pilot studies to evaluate a peer-led, culturally-tailored DSMES intervention for Black men with T2D. The initial pilot (MenD I) was a 3-month randomized trial that demonstrated the peer-led intervention yielded significant benefits in self-management behaviors, with participants who attended at least half the sessions experiencing substantial reductions in diabetes-related emotional distress (16). Building on the lessons from MenD I, MenD II further refined the intervention in several ways: the study period was extended to 15 months with a longer follow-up, the sample size was increased, and the peer leader training curriculum was adapted to incorporate examples and discussion content directly relevant to the experiences of Black men with diabetes. A full description of the adaptation process will be reported in a forthcoming manuscript. In this brief research report, we present the findings from MenD II, focusing on the feasibility and preliminary effectiveness of the enhanced peer-led support model.

## 2 Method

### 2.1 Study design and procedures

We conducted a 15-month peer-led pilot randomized clinical trial (RCT) from July 2021 through January 2023. Data were collected at baseline (T0), three (T1), nine (T2), and 15 (T3) months. A total of 60 Black men were recruited to account for 20% overall attrition, resulting in a final sample size of 48 men. Participants were randomly assigned to the adapted PLDSMS group (n=24) or to the enhanced usual care (EUC) group (n=24). Participants were randomly assigned using a manual randomization procedure conducted in Microsoft Excel upon completion of the baseline assessment. Participants were informed of their group assignment following randomization; blinding of participants or assessors was not implemented given the nature of the intervention. Our sample size followed guidance in the literature for ensuring adequate power for subsequent intervention trials (38).

Three peer leaders were recruited, trained, and assigned to co-facilitate the PLDSMS sessions across two groups of 12 participants each. Following an unforeseen personal circumstance that required one peer leader to discontinue participation during the intervention period, the two remaining peer leaders assumed facilitation responsibilities across all groups for the remainder of the program. Peer leaders were Black men with lived experience managing type 2 diabetes. Inclusion criteria for peer leaders required that candidates be Black/African American males, age 21 or older, with a T2D diagnosis of at least one year, a minimum 8th grade education, willing to commit to 30 hours of training, actively working on their own self-management goals, under the care of a physician for diabetes, and willing to serve in a peer leader role. Peer leader training spanned three months and totaled 30 hours, covering facilitation skills, coping strategies, and empowerment-based communication. Content was adapted to reflect men’s health issues and the lived experiences of Black men with diabetes, and was led by two Certified Diabetes Care and Education Specialists (CDCES) with prior experience developing and implementing the curriculum. To assess treatment fidelity, a checklist evaluated peer leader adherence to three core competency domains: empowerment-based emotional response, group facilitation of the five-step I-SMART problem-solving process, and diabetes knowledge, with two research team members serving as independent raters. Prior to delivering sessions, all three peer leaders completed the training with near-full attendance and achieved passing scores on both competency components; those who scored below threshold on the emotional response domain were offered one revision opportunity and achieved passing scores upon revision. To support ongoing fidelity during the DSMS phase, peer leaders participated in three booster sessions with the CDCES, which reinforced facilitation skills, including emotional exploration, open-ended questioning, and application of the I-SMART process and provided structured opportunities to reflect on implementation challenges. Three DSMS sessions were also randomly selected and recorded to be reviewed by the research team. During the first three months, participants in the PLDSMS arm received 10 hours of virtually delivered DSME, followed by six months of monthly 90-minute PLDSMS sessions. After DSMS concluded, both participants and peer leaders transitioned to six months of ongoing support. Participants in the enhanced usual care (EUC) control group received only the 10-hour virtual DSME over three months, with no DSMS or peer leader support. The EUC condition was selected to isolate intervention effects beyond diabetes education alone, address ethical concerns about a no-treatment control, and account for improvements attributable to program participation and attention. All DSME sessions were led by a CDCES. Upon completion of each assessment (baseline, 3, 9, and 15 months), all participants received $50 per assessment, for a total possible compensation of $200. Additional information on the intervention design is described elsewhere (39). The University of Michigan Medical School Institutional Review Board (IRBMED) HUM00200469 approved the study protocol.

### 2.2 Participants

Eligibility criteria included having T2D for at least six months, being a resident of Metro Detroit, being at least 21 years of age, currently seeing a physician for diabetes care, having access to transportation, and being a member of or regularly attending the participating church where recruitment took place. Exclusion criteria included being non-ambulatory, having serious health conditions, psychiatric illnesses requiring hospitalization, or serious diabetes complications that would negatively interfere with participation.

### 2.3 Measures

Primary outcomes included HbA1c and diabetes self-management behaviors. HbA1c was measured using the DCA 2000 point-of-care testing instrument. Diabetes self-management was measured using the 8-item Perceived Diabetes Self-Management Scale (PDSMS) (40), with summed scores ranging from 8 to 40 and higher scores representing greater self-effectiveness. Secondary outcomes included perceived social support measured using the Duke-UNC Functional Social Support Questionnaire (41, 42) and diabetes distress measured using the Type 2 Diabetes Distress Assessment Tool (43), which assesses core distress and distress from seven specific sources including healthcare provider relationships.

### 2.4 Statistical analysis

A longitudinal mixed-effects linear regression model, adjusted for income, year of diabetes diagnosis, and marital status, was used to compute differences in mean scores across outcomes between groups over time. The Kenward-Roger method estimated standard errors for fixed effects. REML estimation was used to adjust for data missing at random. Fisher’s Exact Test assessed demographic differences between those with optimal (HbA1c <7%) and less than optimal (≥7%) blood glucose levels. Statistical significance was defined as p<0.05. SAS version 9.4 was used for all analyses. Study data were collected using REDCap electronic data capture tools (44).

## 3 Results

**Figure 1:**
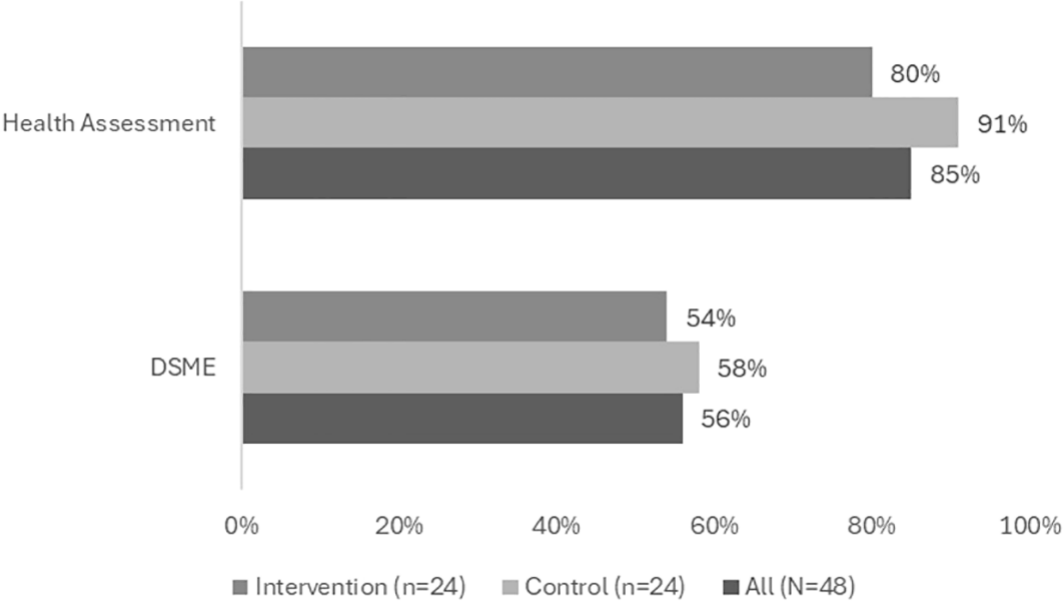
Health assessment and DSME completion rates by group. Completion rates are shown for the Intervention group (n=24), Control group (n=24), and all participants (N=48).

The study demonstrated feasibility with successful recruitment of 60 participants and moderate-to-high retention rates throughout the study period, with greater than 80% of randomized participants completing the 15-month follow-up assessment (**Figure 1**). The clinical and psychosocial outcomes reported below should be interpreted in the context of a pilot study that was not powered to detect statistically significant differences.

Participants and peer leaders (N = 51) ranged in age from 41–80 years (mean: 63 years; SD 9.3) (**Table 1**). Many had at least a bachelor’s degree (n=23; 45.1%), were married (n=23; 45.1%), and were enrolled in Medicare (n=22; 43.1%). HbA1c values ranged from 5.4%-13.4% (mean: 6.8%; SD 1.4). Mean diabetes self-management, social support, and core T2D distress scores were 30.3 (SD 6.3), 39.9 (SD 9.6), and 1.9 (SD 1.0), respectively. There was a statistically significant difference in marital status (p=0.0166) and diabetes self-management scores (p=0.0183) between intervention and EUC groups at baseline.

**Table 1:** Demographic characteristics of participants and peer leaders at baseline.

Characteristic	Participants and peer leaders (N = 51)	Peer leaders (n=3)	Intervention (n=24)	Enhanced usual care (n=24)	p-value
Age (years)*					0.4810
Mean (SD)	63.25 (9.35)	65.67 (7.37)	64.08 (9.64)	62.13 (9.46)	
Range	41 - 80	60 – 74	41 - 78	46 - 80	
Years of formal schooling completed†					0.6431
Some high school	4 (7.84)	--	2 (8.33)	2 (8.33)	
High school graduate/GED	5 (9.80)	--	3 (12.50)	2 (8.33)	
Some college or technical school	19 (37.25)	2 (66.67)	6 (25.00)	11 (45.83)	
College graduate (bachelor’s degree)	11 (21.57)	--	6 (25.00)	5 (20.83)	
Graduate degree	12 (23.53)	1 (33.33)	7 (29.17)	4 (16.67)	
Employment Status†					0.4648
Full-time (≥35 hours/week)	14 (27.45)	--	5 (20.83)	9 (37.50)	
Part-time (<35 hours/week)	6 (11.76)	--	2 (8.33)	4 (16.67)	
Retired	24 (47.06)	3 (100)	12 (50.00)	9 (37.50)	
Disabled, not able to work	6 (11.76)	--	4 (16.67)	2 (8.33)	
Other	1 (1.96)	--	1 (4.17)	--	
Marital Status†					**0.0166‡**
Never Married	7 (13.73)	1 (33.33)	6 (25.00)	--	
Married	23 (45.10)	2 (66.67)	9 (37.50)	12 (50.00)	
Separated/Divorced	18 (35.29)	--	9 (37.50)	9 (37.50)	
Widowed	3 (5.88)	--	--	3 (12.50)	
Annual Household Income ($)†					0.4029
<20,000	11 (21.57)	1 (33.33)	5 (20.83)	5 (20.83)	
20,000-39,999	11 (21.57)	--	7 (29.17)	4 (16.67)	
40,000-59,999	10 (19.61)	1 (33.33)	2 (8.33)	7 (29.17)	
60,000-69,999	6 (11.76)	--	4 (16.67)	2 (8.33)	
≥70,000	13 (25.49)	1 (33.33)	6 (25.00)	6 (25.00)	
Insurance Type (select all that apply)					
Individual plan†	7 (13.73)	--	4 (16.67)	3 (12.50)	1.0000
Group plan through employer§	21 (41.18)	1 (33.33)	8 (33.33)	12 (50.00)	0.2416
Medicaid§	13 (25.49)	1 (33.33)	8 (33.33)	4 (16.67)	0.1824
Medicare§	22 (43.14)	2 (66.67)	11 (45.83)	9 (37.50)	0.5582
Hemoglobin A1c*					0.1670
Mean (SD)	6.8 (1.39)	6.5 (0.76)	6.5 (0.96)	7.1 (1.75)	
Range	5.4 – 13.4	6.0 – 7.4	5.4 – 8.8	5.5 – 13.4	
Diabetes Self-Management, Mean (SD)*||	30.25 (6.30)	33.33 (5.86)	32.21 (4.05)	27.92 (7.47)	**0.0183‡**
Social Support, Mean (SD)*≠	39.86 (9.61)	36.67 (17.93)	39.92 (9.26)	40.21 (9.25)	0.9136
Distress (Core), Mean (SD)*a	1.93 (0.97)	1.91 (0.94)	1.96 (0.94)	1.90 (1.05)	0.8141
Distress (Source), Mean (SD)*b					
Hypoglycemia	1.64 (0.98)	1.44 (0.77)	1.71 (1.11)	1.60 (0.89)	0.7036
Long-term Health	2.08 (1.01)	1.56 (0.38)	2.10 (1.04)	2.13 (1.04)	0.9267
Healthcare Provider	1.65 (0.96)	1.00 (0)	1.74 (1.04)	1.64 (0.93)	0.7346
Interpersonal Issues	1.65 (0.86)	1.00 (0)	1.76 (0.95)	1.63 (0.79)	0.5843
Shame/Stigma	1.33 (0.58)	1.00 (0)	1.39 (0.73)	1.32 (0.44)	0.6919
Healthcare Access	1.65 (0.83)	1.33 (0.58)	1.61 (1.00)	1.74 (0.66)	0.6074
Management Demands	2.12 (0.95)	2.00 (0.88)	2.07 (0.95)	2.18 (0.99)	0.6935

*Significance testing performed using the independent t test.

^†^Significance testing performed using the Fisher’s exact test.

^‡^Significant at the p<0.05 level.

^§^Significance testing performed using the Chi-square test.

^||^Scores range from 8 to 40, with higher scores signifying greater self-efficacy in diabetes self-management.

^≠^Scores range from 8 to 48, with higher scores indicating higher levels of perceived social support.

^a^Scores range from 8 to 40, with higher scores indicating higher levels of core distress.

^b^Higher scores indicate greater levels of distress related to the specific source.

Reported as “n” and prevalence (%).

Peer leaders (n=3) are included in the “Participants and Peer Leaders” column totals but were not randomized to intervention or control conditions.

Bold values indicate statistical significance at p<0.05.

### 3.1 HbA1c

There was no statistically significant difference in HbA1c mean values over time between the intervention and EUC groups (p=0.32) (**Table 2**). Irrespective of treatment group, from baseline to three months, HbA1c mean values increased by 0.10% (0.28 SE; p=0.71). From baseline to nine months, mean values increased by 0.02% (0.31 SE; p=0.94), and from baseline to 15 months, mean HbA1c values increased by 0.18% (0.30 SE; p=0.54).

**Table 2:** Longitudinal mixed-effects linear regression model: intervention effect on primary and secondary outcomes, N = 48.

Outcome	Unadjusted				Outcome	Adjusted*			
	Time Point	B	SE	p-value		Time Point	B	SE	p-value
HbA1c	Overall effect (group*time)			0.8116	HbA1c	Overall effect (group*time)			0.3226
	Baseline (ref)					Baseline (ref)			
	T1	0.002%	0.24	0.9924		T1	0.10%	0.28	0.7125
	T2	-0.04%	0.27	0.8913		T2	0.02%	0.31	0.9372
	T3	0.11%	0.28	0.6905		T3	0.18%	0.30	0.5447
Diabetes Self-Management	Overall effect (group*time)			0.0668	Diabetes Self-Management	Overall effect (group*time)			0.0335†
	Baseline (ref)					Baseline (ref)			
	T1	-0.99	1.58	0.5314		T1	5.36	2.30	0.0248
	T2	-0.15	1.66	0.9294		T2	1.09	2.55	0.6731
	T3	-1.50	1.35	0.2709		T3	3.85	2.26	0.0967
Social Support	Overall effect (group*time)			0.9904	Social Support	Overall effect (group*time)			0.9911
	Baseline (ref)					Baseline (ref)			
	T1	1.77	1.52	0.2556		T1	1.48	1.55	0.3492
	T2	-1.19	1.97	0.5491		T2	-0.98	2.16	0.6518
	T3	0.48	1.50	0.7521		T3	0.99	1.70	0.5636
Diabetes Distress (Core)	Overall effect (group*time)			0.1719	Diabetes Distress (Core)	Overall effect (group*time)			0.3488
	Baseline (ref)					Baseline (ref)			
	T1	-0.05	0.15	0.7279		T1	0.07	0.17	0.6977
	T2	-0.36	0.18	0.0488		T2	-0.23	0.19	0.2459
	T3	-0.24	0.15	0.1161		T3	-0.02	0.17	0.8878
Diabetes Distress (Source - Hypoglycemia)	Overall effect (group*time)			0.1402	Diabetes Distress (Source - Hypoglycemia)	Overall effect (group*time)			0.0778
	Baseline (ref)					Baseline (ref)			
	T1	0.03	0.14	0.8258		T1	0.07	0.14	0.6461
	T2	-0.10	0.16	0.5591		T2	-0.15	0.16	0.3761
	T3	-0.10	0.14	0.4638		T3	-0.08	0.15	0.5830
Diabetes Distress (Source - Long-term Health)	Overall effect (group*time)			0.4819	Diabetes Distress (Source - Long-term Health)	Overall effect (group*time)			0.3818
	Baseline (ref)					Baseline (ref)			
	T1	-0.30	0.16	0.0657		T1	-0.31	0.16	0.0690
	T2	-0.32	0.20	0.1283		T2	-0.40	0.25	0.1136
	T3	-0.45	0.21	0.0384		T3	-0.35	0.20	0.0911
Diabetes Distress (Source - Healthcare Provider)	Overall effect (group*time)			0.2740	Diabetes Distress (Source - Healthcare Provider)	Overall effect (group*time)			0.2159
	Baseline (ref)					Baseline (ref)			
	T1	-0.40	0.15	0.0120		T1	-0.36	0.16	0.0339
	T2	-0.40	0.19	0.0386		T2	-0.5	0.22	0.0281
	T3	-0.49	0.14	0.0010		T3	-0.54	0.15	0.0012
Diabetes Distress (Source - Interpersonal Issues)	Overall effect (group*time)			0.5294	Diabetes Distress (Source - Interpersonal Issues)	Overall effect (group*time)			0.5488
	Baseline (ref)					Baseline (ref)			
	T1	-0.29	0.12	0.0244		T1	-0.24	0.13	0.0746
	T2	-0.37	0.14	0.0149		T2	-0.36	0.18	0.0544
	T3	-0.35	0.16	0.0339		T3	-0.32	0.19	0.1107
Diabetes Distress (Source - Shame/Stigma)	Overall effect (group*time)			0.5949	Diabetes Distress (Source - Shame/Stigma)	Overall effect (group*time)			0.5462
	Baseline (ref)					Baseline (ref)			
	T1	-0.01	0.08	0.8885		T1	-0.04	0.07	0.5996
	T2	-0.14	0.10	0.1647		T2	-0.16	0.11	0.1538
	T3	-0.17	0.08	0.0380		T3	-0.25	0.10	0.0158
Diabetes Distress (Source - Healthcare Access)	Overall effect (group*time)			0.8673	Diabetes Distress (Source - Healthcare Access)	Overall effect (group*time)			0.4315
	Baseline (ref)					Baseline (ref)			
	T1	-0.18	0.14	0.1927		T1	-0.12	0.15	0.4334
	T2	-0.10	0.12	0.4162		T2	0.05	0.12	0.6917
	T3	-0.12	0.12	0.3193		T3	0.14	0.14	0.3449
Diabetes Distress (Source - Management Demands)	Overall effect (group*time)			0.1162	Diabetes Distress (Source - Management Demands)	Overall effect (group*time)			0.2009
	Baseline (ref)					Baseline (ref)			
	T1	-0.28	0.15	0.0639		T1	-0.18	0.17	0.2903
	T2	-0.46	0.19	0.0215		T2	-0.31	0.21	0.1380
	T3	-0.33	0.17	0.0596		T3	-0.04	0.20	0.8578

*Adjusted for income, year of diabetes diagnosis, and marital status .

^†^Difference in mean scores between the intervention and Enhanced Usual Care (EUC) groups over time (reference group = intervention) - Significant at the 0.05 level.

Estimation Method: REML (residual/restricted maximum likelihood).

Fixed Effects SE Method: Kenward-Roger.

At T1, both groups received the same content

Bold values indicate statistical significance at p<0.05.

### 3.2 Perceived diabetes self-management

**Figure 2:**
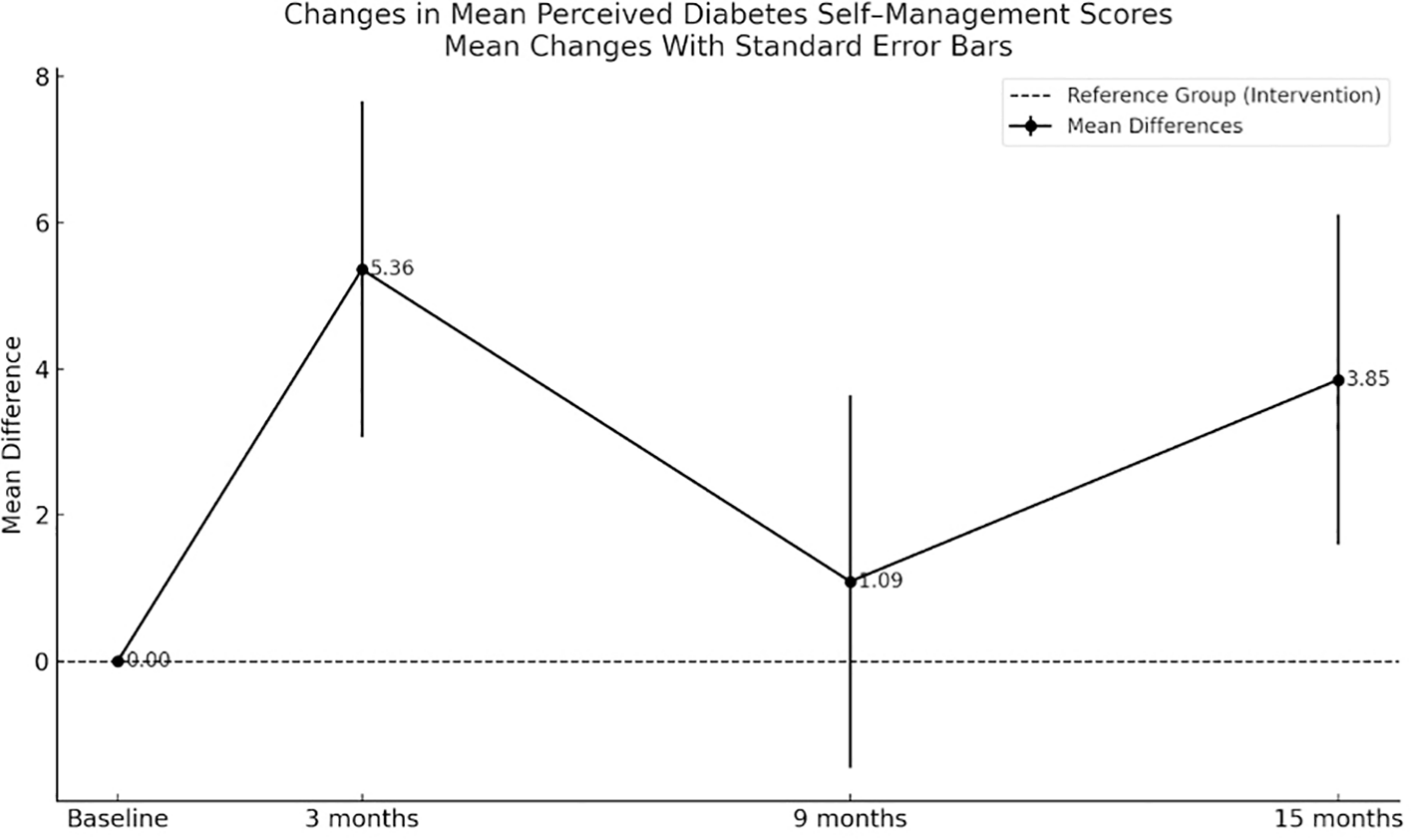
Changes in mean perceived diabetes self-management scores with standard error bars. Significant differences were observed between the intervention and EUC control groups over time (p=0.0335). N=48.

Changes in perceived diabetes self-management scores significantly differed over time between the intervention and EUC groups (p=0.03) (**Figure 2**). On average, those in the EUC group had scores that were 5.36 points higher from baseline to three months than those in the intervention group (2.30 SE; p=0.02), with differences in score changes between groups narrowing at nine months (B = 1.09; 2.55 SE; p=0.67).

### 3.3 Social support and diabetes distress

There was no significant difference in changes in mean social support scores over time between groups (p=0.99). Changes in core diabetes distress scores over time between groups were also not significant (p=0.35). However, irrespective of treatment group, there were statistically significant changes over time in diabetes distress scores related to healthcare provider relationships. Distress scores steadily decreased from baseline to three months (B=-0.36; 0.16 SE; p=0.03), baseline to nine months (B=-0.50; 0.22 SE; p=0.03), and from baseline to 15 months (B=-0.54; 0.15 SE; p=0.001).

## 4 Discussion

This pilot study evaluated a 15-month culturally tailored peer-led diabetes self-management support (PLDSMS) program for Black men, plus standard diabetes self-management education (DSME) compared to DSME alone. We found no significant between-group differences in glycemic control, likely due in part to relatively well-controlled baseline HbA1c levels (around 6.8%). Our earlier pilot (MenD I) demonstrated that peer-led support could yield behavioral improvements even with brief intervention duration (16). The current study similarly showed stable HbA1c but mixed psychosocial findings. Perceived diabetes self-management showed a significant group × time interaction (p=0.03), though the pattern was complex: the control group showed greater improvements at 3 months, with group differences narrowing by 9 months. Importantly, diabetes distress related to healthcare providers declined significantly over time in both groups (baseline to 15 months: B=-0.54, p=0.001), suggesting that even standard DSME with a CDCES may help alleviate provider-related stress.

The feasibility demonstrated in this study builds upon growing evidence that culturally adapted, gender-concordant interventions can successfully engage Black men with diabetes. Unlike traditional diabetes programs where most peer supporters and participants have been women, our intervention leveraged male peers and male-only groups, directly addressing the sociocultural context of Black men’s health (16, 39, 45). This approach resonates with emerging research showing that Black men respond especially well to peer support delivered by those with shared life experience (15).

While our study did not demonstrate superior effectiveness on primary outcomes, the complex trajectory of self-management scores warrants consideration. The pattern may reflect an initial short-term improvement following DSME alone, characterized by early gains that attenuate in the absence of ongoing peer support, while the peer-led component may help sustain motivation over time. Future analyzes with longer follow-up could test this hypothesis. A systematic review confirms that robust doctor–patient communication is linked to better adherence and clinical outcomes in chronic illness. Fostering adaptive, patient-centered communication can empower patients and reduce their diabetes-related distress.

Notably, this study did not yield significant improvements in perceived social support (p=0.99). Monthly online meetings may have been insufficient to forge deep interpersonal connections. Although a randomized trial found that telehealth-based education was comparable to in-person education for glycemic outcomes, researchers emphasized the need to optimize the timing and intensity of virtual contact (31). In future iterations, increasing contact frequency (biweekly or weekly check-ins), facilitating peer-to-peer communication between sessions, or using a hybrid model could strengthen group cohesion and perceived support.

### 4.1 Scalability and implementation implications

The use of a virtual, peer-led model offers promise for broader dissemination. Virtual delivery greatly expands reach by overcoming geographic and scheduling barriers, a critical advantage for engaging Black men who often face work, transportation, and mistrust barriers in healthcare (16, 39, 45). Studies have demonstrated that online diabetes self-management interventions can significantly improve glycemic stability and be cost-effective (46). Community health centers, churches, fraternities, and workplaces could serve as recruitment hubs, while trained Black male peer coaches facilitate groups via video or phone. Our findings will inform a future fully powered trial and have near-term relevance for practice. A peer-led approach grounded in Self-Determination Theory and patient empowerment can be a scalable model to improve chronic disease outcomes in this underserved population, as it cultivates autonomy, competence, and relatedness, the key drivers of intrinsic motivation for sustained self-care (47, 48).

### 4.2 Strengths and limitations

Key strengths include focus on a historically underrepresented group in diabetes research and deliberate grounding in Self-Determination Theory and the patient empowerment paradigm. The male peer-led, culturally tailored design addresses well-documented sociocultural barriers. Another strength is the pragmatic use of a virtual platform, which improves accessibility and scalability.

However, several limitations temper our conclusions. The sample size (48 participants) was small and the study was underpowered to detect modest clinical changes. Many participants had relatively well-controlled diabetes (mean HbA1c 6.8% at baseline), leaving little room for HbA1c improvement. Importantly, with the study being underpowered, the null HbA1c finding does not suggest a lack of intervention effect. Baseline PDSMS scores were not equivalent across intervention and EUC groups, which may have influenced observed between−group differences. Although baseline imbalances can occur by chance in small randomized trials, this remains a limitation of the study. The study took place in a single region (Metro Detroit) with volunteers largely connected to community organizations, which may limit generalizability. Data collection began during the recovery phase of the COVID-19 pandemic, when external stressors might have influenced participants’ behaviors. The virtual format may have influenced group dynamics, the lack of face-to-face interaction could reduce perceived benefits of peer bonding. Finally, reliance on self-reported measures introduces the possibility of response bias.

In summary, the MenD II pilot demonstrates that a peer-led, culturally tailored diabetes self-management program for Black men is feasible, as evidenced by high retention rates (>80% at 15 months) and successful recruitment. While the intervention did not demonstrate superior effectiveness compared to standard DSME on glycemic control or most psychosocial outcomes, the study successfully established that this approach can engage and retain Black men, a population historically difficult to reach in diabetes interventions. These insights will guide refinements for future research. More broadly, our findings add to the evidence that empowering, community-driven approaches can successfully engage Black men in chronic disease management.

## Data Availability

The raw data supporting the conclusions of this article will be made available by the authors, without undue reservation.

## References

[1] Centers for Disease Control and Prevention . Type 2 diabetes (2024). Available online at: https://www.cdc.gov/diabetes/about/about-type-2-diabetes.html (Accessed November 1, 2025).

[2] Centers for Disease Control and Prevention . National diabetes statistics report. Available online at: https://www.cdc.gov/diabetes/data/statistics-report/index.html (Accessed November 1, 2025).

[3] American Diabetes Association . The burden of diabetes in Michigan. Available online at: https://diabetes.org/sites/default/files/2021-11/ADV_2021_State_Fact_sheets_Michigan_rev.pdf (Accessed November 1, 2025).

[4] Zakaria NI Tehranifar P Laferrère B Albrecht SS . Racial and ethnic disparities in glycemic control among insured US adults. JAMA Netw Open. (2023) 6:e2336307. doi: 10.1001/jamanetworkopen.2023.36307. PMID: 37796503 PMC10556965

[5] Hawkins J Watkins DC Kieffer E Spencer M Espitia N Anderson M . Psychosocial factors that influence health care use and self-management for African American and Latino men with type 2 diabetes: An exploratory study. J Mens Stud. (2015) 23:161–76. doi: 10.1177/1060826515582495

[6] Hawkins J Watkins DC Kieffer E Spencer M Piatt G Nicklett EJ . An exploratory study of the impact of gender on health behavior among African American and Latino men with type 2 diabetes. Am J Mens Health. (2017) 11:344–56. doi: 10.1177/1557988316681125. PMID: 27923970 PMC5675282

[7] Hawkins JM . Type 2 diabetes self-management in non-Hispanic black men: A current state of the literature. Curr Diabetes Rep. (2019) 19:1–6. doi: 10.1007/s11892-019-1131-8. PMID: 30741346

[8] Powell W Adams LB Cole-Lewis Y Agyemang A Upton RD . Masculinity and race-related factors as barriers to health help-seeking among African American men. Behav Med. (2016) 42:150–63. doi: 10.1080/08964289.2016.1165174. PMID: 27337619 PMC4979354

[9] Liburd LC Namageyo-Funa A Jack L . Understanding "masculinity" and the challenges of managing type-2 diabetes among African-American men. J Natl Med Assoc. (2007) 99:550. doi: 10.2337/diaspect.17.4.219 17534013 PMC2576079

[10] Bruce MA Griffith DM Thorpe RJ . Stress and the kidney. Adv Chronic Kidney Dis. (2015) 22:46–53. doi: 10.1053/j.ackd.2014.06.008. PMID: 25573512 PMC4871619

[11] Griffith DM . I AM a Man": Manhood, minority men's health and health equity. Ethn Dis. (2015) 25:287. doi: 10.18865/ed.25.3.287. PMID: 26672674 PMC4671414

[12] Pittman DM Brooks JJ Kaur P Obasi EM . The cost of minority stress: Risky alcohol use and coping-motivated drinking behavior in African American college students. J Ethn Subst Abuse. (2019) 18:257–78. doi: 10.1080/15332640.2017.1336958. PMID: 28708010 PMC6070424

[13] Davis J Fischl AH Beck J Browning L Carter A Condon JE . 2022 National standards for diabetes self-management education and support. Sci Diabetes Self Manag Care. (2022) 48:44–59. doi: 10.1177/26350106211072203. PMID: 35049403

[14] Haas L Maryniuk M Beck J . National standards for diabetes self-management education and support. Diabetes Care. (2012) 35:2393–401. doi: 10.1177/0145721712455997. PMID: 22995096 PMC3476915

[15] Shiyanbola OO Maurer M Schwerer L Sarkarati N Wen MJ Salihu EY . A culturally tailored diabetes self-management intervention incorporating race-congruent peer support to address beliefs, medication adherence and diabetes control in African Americans: a pilot feasibility study. Patient Prefer Adherence. (2022) 16:2893–912. doi: 10.2147/ppa.s384974. PMID: 36317056 PMC9617564

[16] Ewen AM Hawkins JM Kloss KA Nwankwo R Funnell MM Sengupta S . The Michigan Men's diabetes project randomized clinical control trial: A pilot/feasibility study of a peer-led diabetes self-management and support intervention for black men with type 2 diabetes. Am J Mens Health. (2024) 18:15579883241258318. doi: 10.1177/15579883241258318. PMID: 38879823 PMC11181889

[17] Crabtree K Sherrer N Rushton T Willig A Agne A Shelton T . Diabetes connect: African American men's preferences for a community-based diabetes management program. Diabetes Educ. (2015) 41:118–26. doi: 10.1177/0145721714557043 PMC5166559 25367259

[18] Griffith DM Bergner EM Cornish EK McQueen CM . Physical activity interventions with African American or Latino men: A systematic review. Am J Mens Health. (2018) 12:1102–17. doi: 10.1177/1557988318763647. PMID: 29557237 PMC6131438

[19] Griffith DM Allen JO Johnson-Lawrence V Langford A . Men on the move: a pilot program to increase physical activity among African American men. Health Educ Behav. (2014) 41:164–72. doi: 10.1177/1090198113496788 PMC4511956 23918885

[20] Wippold GM Frary SG Abshire D Wilson DK . Peer-to-peer health promotion interventions among African American men: a scoping review protocol. Syst Rev. (2021) 10:184. doi: 10.1186/s13643-021-01737-y. PMID: 34154638 PMC8218504

[21] Balls-Berry J Watson C Kadimpati S Crockett A Mohamed EA Brown I . Black men's perceptions and knowledge of diabetes: A church-affiliated barbershop focus group study. J Racial Ethn Health Disparities. (2015) 2:465–72. doi: 10.1007/s40615-015-0094-y. PMID: 26594612 PMC4651172

[22] Moore N Wright M Gipson J Jordan G Harsh M Reed D . A survey of African American men in Chicago barbershops: Implications for the effectiveness of the barbershop model in the health promotion of African American men. J Community Health. (2016) 41:772–9. doi: 10.1007/s10900-016-0152-3. PMID: 26831485

[23] Murphy AB Moore NJ Wright M Gipson J Keeter M Cornelious T . Alternative locales for the health promotion of African American men: a survey of African American men in Chicago barbershops. J Community Health. (2017) 42:139–46. doi: 10.1007/s10900-016-0240-4. PMID: 27651166 PMC5839325

[24] Wade JM Dillon H Robinson K Ongeri EM Rivas KT Cook M . People gather here for open conversations, and health should be in our open conversations": promoters of black men's engagement in diabetes screenings at local barbershops. J Racial Ethn Health Disparities. (2024) 11:1260–8. doi: 10.1007/s40615-023-01605-6. PMID: 37095289 PMC12459871

[25] Afshar R Tang TS Askari AS Sidhu R Brown H Sherifali D . Peer support interventions in type 2 diabetes: Review of components and process outcomes. J Diabetes. (2020) 12:315–38. doi: 10.1111/1753-0407.12999. PMID: 31639255

[26] Arévalo Avalos MR Patel A Duru H Shah S Rivera M Sorrentino E . Implementation of a technology-enabled diabetes self-management peer coaching intervention for patients with poorly controlled diabetes: quasi-experimental case study. JMIR Diabetes. (2024) 9:e54370. doi: 10.2196/54370. PMID: 39405529 PMC11522654

[27] Presley C Agne A Shelton T Oster R Cherrington A . Mobile-enhanced peer support for African Americans with type 2 diabetes: a randomized controlled trial. J Gen Intern Med. (2020) 35:2889–96. doi: 10.1007/s11606-020-06011-w. PMID: 32700215 PMC7572958

[28] Hulbert L Mensa-Wilmot Y Rutledge S Owens-Gary M Skeete R Cannon MJ . Interests and preferences in programs to improve health among men with or at risk for type 2 diabetes in racial and ethnic minority groups, 2019. Prev Chronic Dis. (2025) 22:E04. doi: 10.5888/pcd22.240268. PMID: 39784112 PMC11721013

[29] Hawkins JM Ewen AM Funnell M Nwankwo R Piatt G . Lessons learned in recruiting and retaining Black men in behavioral diabetes intervention research. Contemp Clin Trials Commun. (2025) 45:101487. doi: 10.1016/j.conctc.2025.101487. PMID: 40420865 PMC12104708

[30] Piatt G Provenzano AM Nwankwo R Hall D Kloss KA Hawkins JM . 62-OR: Fostering sustainability through diabetes self-management support in African-American churches: Results of the Praise Diabetes project. Diabetes. (2021) 70:62–OR. doi: 10.2337/db21-62-or 33115827

[31] Ballesta S Chillarón JJ Inglada Y Climent E Llauradó G Pedro-Botet J . Telehealth model versus in-person standard care for persons with type 1 diabetes treated with multiple daily injections: an open-label randomized controlled trial. Front Endocrinol. (2023) 14:1176765. doi: 10.3389/fendo.2023.1176765. PMID: 37441496 PMC10333924

[32] Deci EL Eghari H Patrick BC Leone D . Facilitating internalization: the self determination theory perspective. J Pers. (1994) 62:119–42. doi: 10.1111/j.1467-6494.1994.tb00797.x. PMID: 8169757

[33] Williams GC Freedman ZR Deci EL . Supporting autonomy to motivate patients with diabetes for glucose control. Diabetes Care. (1998) 21:1644–51. doi: 10.2337/diacare.21.10.1644. PMID: 9773724

[34] Williams GC McGregor HA Zeldman A Freedman ZR Deci EL . Testing a self determination theory process model for promoting glycemic control through diabetes self-management. Health Psychol. (2004) 23:58–66. doi: 10.1037/0278-6133.23.1.58. PMID: 14756604

[35] Tang TS Funnell MM Sinco B Spencer MS Heisler M . Peer-led, empowerment-based approach to self-management efforts in diabetes (PLEASED): a randomized controlled trial in an African American community. Ann Fam Med. (2015) 13:S27–35. doi: 10.1370/afm.1819. PMID: 26304969 PMC4648139

[36] Cunningham AT Crittendon DR White N Mills GD Diaz V LaNoue MD . The effect of diabetes self-management education on HbA1c and quality of life in African Americans: a systematic review and meta-analysis. BMC Health Serv Res. (2018) 18:367. doi: 10.1186/s12913-018-3186-7. PMID: 29769078 PMC5956958

[37] Samuel-Hodge CD Keyserling TC France R Ingram AF Johnston LF Davis LP . A church-based diabetes self-management education program for African Americans with type 2 diabetes. Prev Chronic Dis. (2006) 3:A93. doi: 10.1177/0145721709333270. PMID: 16776894 PMC1637801

[38] Bell ML Whitehead AL Julious SA . Guidance for using pilot studies to inform the design of intervention trials with continuous outcomes. Clin Epidemiol. (2018) 10:153–7. doi: 10.2147/clep.s146397. PMID: 29403314 PMC5779280

[39] Hawkins J Sengupta S Kloss K Kurnick K Ewen A Nwankwo R . Michigan men's diabetes project II: Protocol for peer-led diabetes self-management education and long-term support in Black men. PloS One. (2023) 18:e0277733. doi: 10.1371/journal.pone.0277733. PMID: 36862648 PMC9980828

[40] Wallston KA Rothman RL Cherrington A . Psychometric properties of the perceived diabetes self-management scale (PDSMS). J Behav Med. (2007) 30:395–401. doi: 10.1007/s10865-007-9110-y. PMID: 17522972

[41] Broadhead WE Gehlbach SH De Gruy FV Kaplan BH . The Duke-UNC Functional Social Support Questionnaire: Measurement of social support in family medicine patients. Med Care. (1988) 26:709–23. doi: 10.1097/00005650-198807000-00006 3393031

[42] Epino HM Rich ML Kaigamba F Hakizamungu M Socci AR Bagiruwigize E . Reliability and construct validity of three health-related self-report scales in HIV-positive adults in rural Rwanda. AIDS Care. (2012) 24:1576–83. doi: 10.1080/09540121.2012.661840. PMID: 22428702

[43] Polonsky WH Fisher L Hessler D Desai U King SB Perez-Nieves M . Toward a more comprehensive understanding of the emotional side of type 2 diabetes: A re-envisioning of the assessment of diabetes distress. J Diabetes Complications. (2022) 36:108103. doi: 10.1016/j.jdiacomp.2021.108103. PMID: 34916146

[44] Harris PA Taylor R Thielke R Payne J Gonzalez N Conde JG . Research electronic data capture (REDCap), a metadata-driven methodology and workflow process for providing translational research informatics support. J BioMed Inform. (2009) 42:377–81. doi: 10.1016/j.jbi.2008.08.010. PMID: 18929686 PMC2700030

[45] Hawkins J Kloss K Funnell M . Michigan Men's diabetes project (MenD): protocol for a peer leader diabetes self-management education and support intervention. BMC Public Health. (2021) 21:562. doi: 10.1186/s12889-021-10613-2. PMID: 33752609 PMC7983198

[46] Greenwood DA Gee PM Fatkin KJ Peeples M . A systematic review of reviews evaluating technology-enabled diabetes self-management education and support. J Diabetes Sci Technol. (2017) 11:1015–27. doi: 10.1177/1932296817713506. PMID: 28560898 PMC5951000

[47] Tarfa A Salihu EY Xiong P Brewer C Maurer M Liu Y . Participant and group facilitator perspectives on a novel culturally tailored diabetes self-management program for African Americans. BMC Public Health. (2024) 24:3106. doi: 10.1186/s12889-024-20595-6. PMID: 39529036 PMC11552422

[48] Wen MJ Salihu EY Yang C Maurer M Shiyanbola OO . Peer ambassador perspectives in a culturally tailored self-management intervention for African Americans with type 2 diabetes: a qualitative study. Pharmacy. (2024) 12:75. doi: 10.20944/preprints202404.0514.v1 38804467 PMC11130834

